# Supplementary material for: Comprehensive Annotation and Functional Exploration of MicroRNAs in Lettuce
Source: Front Plant Sci. 2021 Dec 24;12:781836. doi: 10.3389/fpls.2021.781836 (PMC8739914; doi:10.3389/fpls.2021.781836)
Supplement: Supplementary file 3 [file Data_Sheet_9.ZIP › GSM2230752.plot/Lsa-miRN42_Lsat_1_v5_gn_3_47821.1_2649_TPlot.pdf]

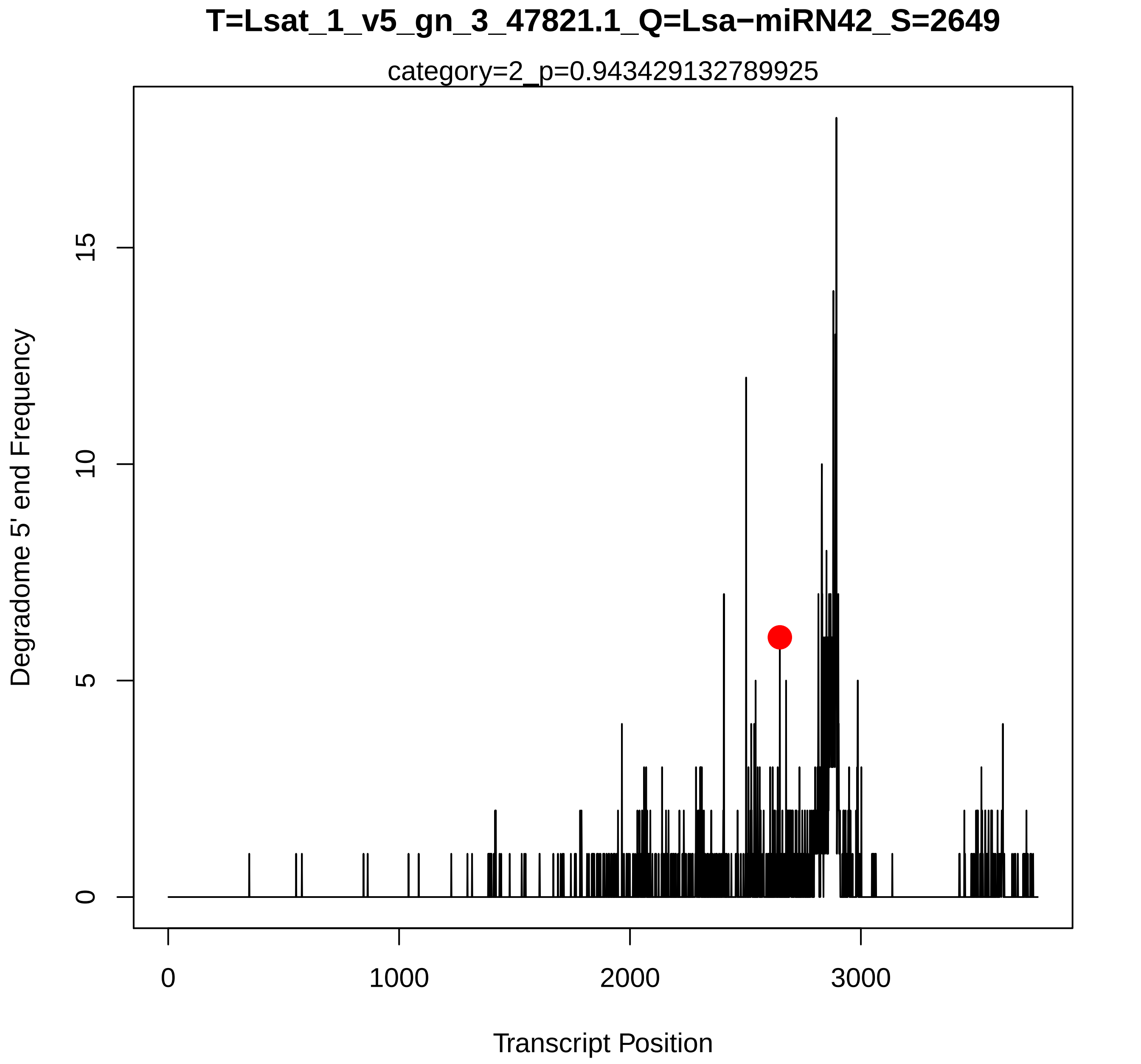
T=Lsat_1_v5_gn_3_47821.1_Q=Lsa−miRN42_S=2649
category=2_p=0.943429132789925
Degradome 5' end Frequency
0
5
10
15
0
1000
2000
3000
Transcript Position
